# Comprehensive Analysis of Prokaryotes in Environmental Water Using DNA Microarray Analysis and Whole Genome Amplification

**DOI:** 10.3390/pathogens2040591

**Published:** 2013-10-30

**Authors:** Takeshi Akama, Akira Kawashima, Kazunari Tanigawa, Moyuru Hayashi, Yuko Ishido, Yuqian Luo, Akihisa Hata, Noboru Fujitani, Norihisa Ishii, Koichi Suzuki

**Affiliations:** 1 Laboratory of Molecular Diagnostics, Department of Mycobacteriology, Leprosy Research Center, National Institute of Infectious Diseases, Tokyo 189-0002, Japan; E-Mails: akama@umich.edu (T.A.); akirak@jichi.ac.jp (A.K.); tanigawa@pharm.teikyo-u.ac.jp (K.T.); mhayashi@rs.kagu.tus.ac.jp (M.H.); yishido@nih.go.jp (Y.I.); yuqianluo31@gmail.com (Y.L); 2 Department of Medical Risk Management, Faculty of Risk Management, Chiba Institute of Science, Faculty of Risk and Crisis Management, Choshi, Chiba 288-0025, Japan; E-Mails: ahata@cis.ac.jp (A.H.); nfujitani@cis.ac.jp (N.F.); 3 Leprosy Research Center, National Institute of Infectious Diseases, Tokyo 189-0002, Japan; E-Mail: norishii@nih.go.jp

**Keywords:** metagenome, microarray, 16S rRNA, WGA, pathogenic bacteria

## Abstract

The microflora in environmental water consists of a high density and diversity of bacterial species that form the foundation of the water ecosystem. Because the majority of these species cannot be cultured *in vitro*, a different approach is needed to identify prokaryotes in environmental water. A novel DNA microarray was developed as a simplified detection protocol. Multiple DNA probes were designed against each of the 97,927 sequences in the DNA Data Bank of Japan and mounted on a glass chip in duplicate. Evaluation of the microarray was performed using the DNA extracted from one liter of environmental water samples collected from seven sites in Japan. The extracted DNA was uniformly amplified using whole genome amplification (WGA), labeled with Cy3-conjugated 16S rRNA specific primers and hybridized to the microarray. The microarray successfully identified soil bacteria and environment-specific bacteria clusters. The DNA microarray described herein can be a useful tool in evaluating the diversity of prokaryotes and assessing environmental changes such as global warming.

## 1. Introduction

Diverse prokaryotic populations, including bacteria and archaea, co-exist in the microflora of various environments such as soil, air and water. Microbes in environmental water constitute 50%–90% of ocean biomass, with 3 × 10^4^ species per liter at a density of 10^4^ to 10^7^ cells per milliliter [1,2]. Therefore, the microflora in environmental water forms the basis of the food chain, and changes in microflora biomass or composition influence the entire water ecosystem.

Composition of the environmental microflora is dependent on various conditions such as temperature, salinity, organic or inorganic nutrients, and human activities [1,3]. *In vitro* culture and isolation of each organism were employed in an attempt to determine the microbial composition of environmental samples. However, it became evident that most environmental bacteria cannot be cultured [4]. As an alternative, genomic DNA was extracted from samples of marine water, fresh water, and soil for the analysis of DNA sequences. The analysis of marine water in the Sargasso Sea found 148 novel phylotypes and 1.2 million genes among 1.6 Gbp of DNA sequence [5]. These metagenomic data were integrated with those of a global ocean sampling expedition that collected samples at 44 sites, from the Atlantic Ocean near the Nova Scotia peninsula in Canada to the Pacific Ocean near French Polynesia via the Gulf of Panama [6]. The 6.3 Gbp of sequence demonstrated taxonomic, subspecies, genetic, and geographical diversity. Comparative metagenomic analysis of samples collected from marine, hyper saline, fresh, and coral reef water uncovered differences between the metabolic profiles in each environment [7].

Taxonomic analysis of metagenomes using DNA microarrays is simpler and more reproducible than sequencing, which is still an expensive and complicated procedure [8]. However, previous 16S rRNA arrays covered only 800 to 9,000 sequences, which were not enough to analyze microflora in environmental metagenomic DNA samples [9].

Using the DNA Data Bank of Japan (DDBJ), which has collected >140,000 16S rRNA sequences, we designed probes for a DNA microarray to detect prokaryotic species. We then applied this array to the metagenomic analysis of microflora from water samples around Japan. Individual pathogenic bacteria were detected, as well as specific prokaryotic clusters that might be associated with the temperature of the water that was sampled.

## 2. Results and Discussion

### 2.1. Sampling of Environmental Water

**Figure 1:**
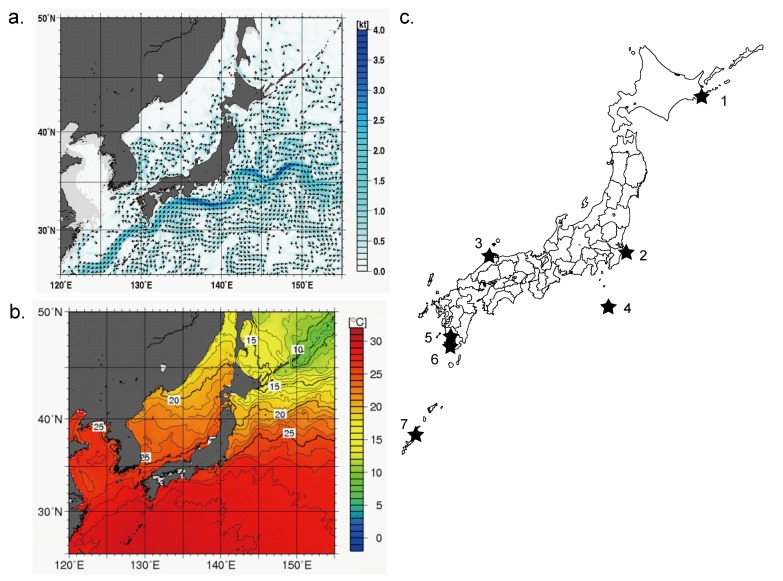
Ocean flow, marine temperature, and sampling sites in Japan. (**a**) Mean sea surface current (direction and knots) for 1–10 August 2009; (**b**) Monthly mean sea surface temperature (°C) in August 2009 obtained from the Japan Meteorological Agency [11]; (**c**) Sampling sites and associated sample numbers (see Table 1).

According to information supplied by the Japan Meteorological Agency [10] the Black Current flows from the south of Japan along the Pacific side of the archipelago and partially into the Sea of Japan (Figure 1a). The frigid Oyashio Current flows from north of Japan (Hokkaido) along the Pacific coast and runs into the Black Current. These two currents create the basis of warm and cold sea surface temperatures around Japan (Figure 1b). Environmental water was collected from seven sites in Japan (Figure 1c). The temperature, pH and amounts of DNA recovered from 1 L of water samples before whole genome amplification (WGA) are shown in Table 1.

**Table 1:** Sampling sites, environmental data and recovered total DNA.

No.	Sampling site	Salinity	Sampling	Temperature (°C)	pH	DNA recovery (μg)
1	Nemuro	Marine	July 28	13.5	7.9	1.10
2	Tonegawa river	Estuarine water	May 23	21.0	8.0	5.60
3	Shimanekaka	Marine	August 18	27.0	8.2	6.20
4	Hachijo	Marine	August 3	28.0	8.3	0.12
5	Kagoshima	Marine	September 6	29.9	8.2	0.24
6	Ikeda lake	Fresh water	August 3	28.8	8.3	0.96
7	Yoron	Marine	September 10	29.5	8.0	0.66

### 2.2. Taxonomic Composition in Each Water Sample

DNA extracted from water samples collected from seven sites around Japan was amplified using WGA (typically 10 to 100-fold amplification was achieved) and subjected to DNA microarray analysis. Since signals from most probes were almost negative, probes that showed top 1,000 signals were subjected for taxonomical analysis (see Section 3.5 Data Analysis of DNA Microarray). 

**Figure 2:**
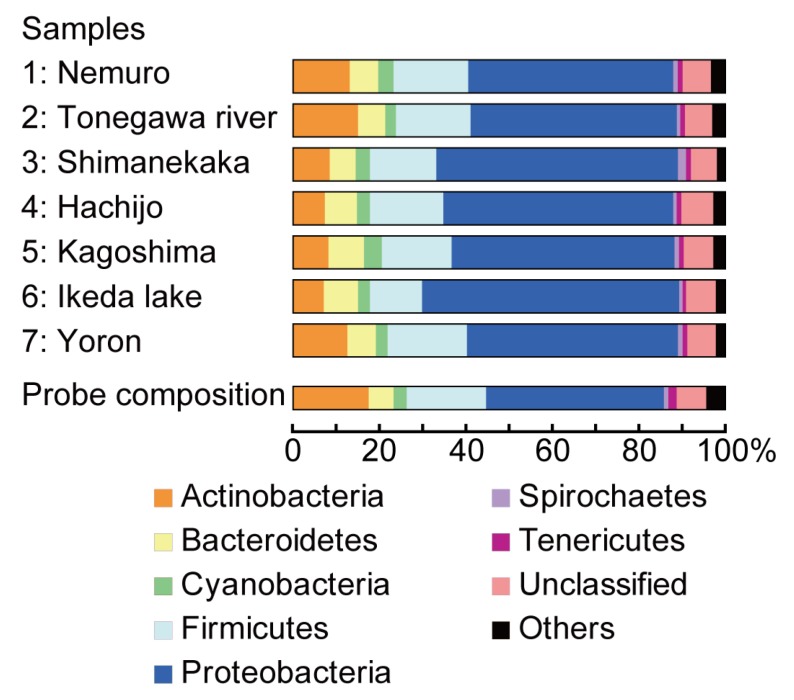
.Microarray data classified at the phylum level. The percentage of each phylum comprising the microarray probes and the top 1,000 target sequences from each samples 1–7. The actual percentage in each phylum including those for classified as “others” were shown in Table S1. *p* = 10^−8^ as evaluated by the proposed algorithm [12].

Phyla distribution was somewhat similar among samples and that of original probe composition (Figure 2 and Table S1), suggesting that this array detected each phylum without having significant difference in affinity and sensitivity of each probe spotted on the array. Sample 1 obtained from northern part of Japan with much lower water temperature (Table 1) showed no significant difference in the taxonomical distribution (Figure 2, Table S1 and Figure S1), suggesting that the local fluctuation of surface temperature may not be the most decisive factor to affect microbiota, and that the most bacterial flora around Japan islands are essentially similar.

Nevertheless, small differences in distribution as shown in the levels of phylum (Figure 2), class, order, family, genus and species (Figure S1) also suggest that some of the prokaryotes are unevenly distributed around Japan. Indeed, sample 6, the only lake sample, showed a unique pattern compared to others (Figure 2 and Figure S1). These results also suggest that although the composition of most bacteria in the environment is similar, this microarray could detect small differences in the prokaryotic species in environmental water. However, it is still difficult to demonstrate clear differences in the proportion of water prokaryotes at the phylum level or at the any level of taxonomy; therefore, we proceeded to perform further identification of some specific bacteria and deeper taxonomy analysis by cluster analysis.

### 2.3. Detection of Pathogenic Bacteria

We examined whether DNA from pathogenic bacteria can be detected in the top 1,000 signals generated in each sample (see Section 3.5 Data Analysis of DNA Microarray). Five species were detected in the seven samples (Table 2). *Aeromonas sobria* [13], *Citrobacter freundii* [14], and *Clostridium perfringens* [15] were detected in multiple samples, while *Mycobacterium gordonae* [16] was only found in sample 7 and *Mycobacterium marinum* [17] in sample 2. These data indicate a possibility that this microarray can be used to screen pathogenic bacteria in the environment, although further confirmation is needed.

**Table 2:** Pathogenic bacteria detected by the DNA microarray.

Species	Sample number						
	**1**	**2**	**3**	**4**	**5**	**6**	**7**
*Aeromonas sobria*	−	+	+	+	+	+	+
*Citrobacter freundii*	−	−	+	+	+	+	+
*Clostridium perfringens*	+	+	+	+	+	+	+
*Mycobacterium gordonae*	−	−	−	−	−	−	+
*Mycobacterium marinum*	−	+	−	−	−	−	−

### 2.4. Detection of Site-Specific Clusters and Species

**Figure 3:**
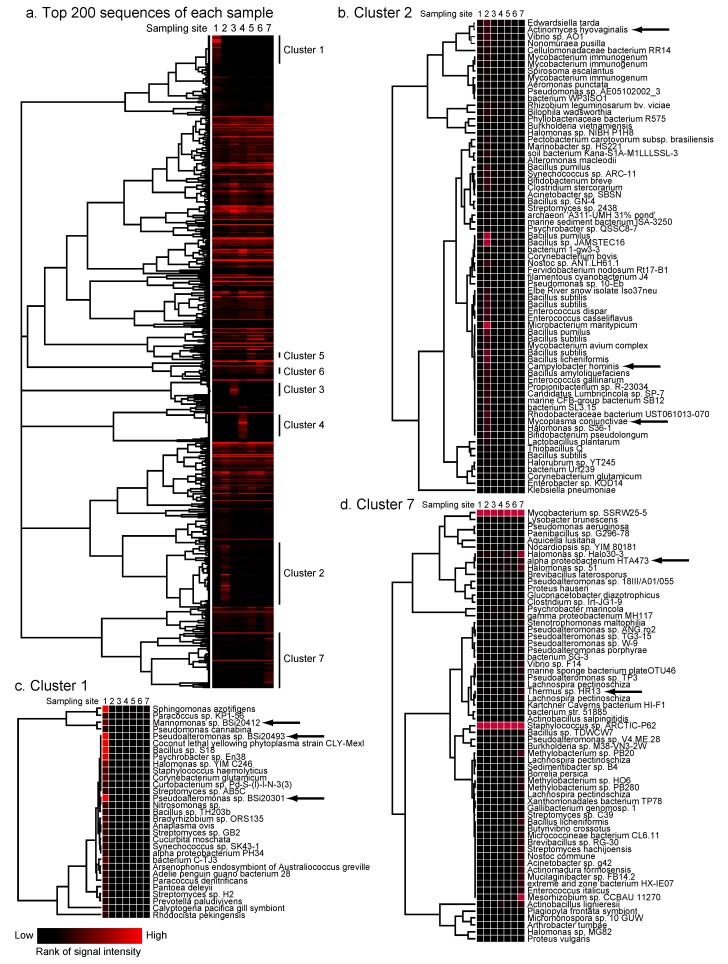
Cluster analysis of sequences detected on the microarray. Sequences with the top 200 signal intensities in each sample were selected. The rank of the signal intensity in each microarray was changed to the reciprocal number, analyzed with Cluster 3.0, and visualized using Treeview 1.0. (**a**) The clusters that exhibited specific, strong signals only in one of the sampling sites 1 to 7 are marked as clusters 1 to 7. The detail of clusters 2 (**b**), 1 (**c**) and 7 (**d**) are shown with species names. Species indicated by arrows are the characteristic types in each cluster.

**Figure 4:**
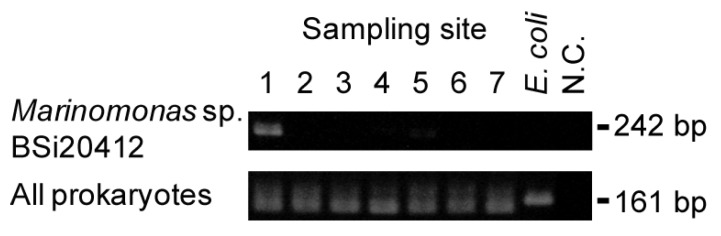
Nested PCR to detect a specific product of *Marinomonas* sp. BSi20412. 16S rRNA sequences of *Marinomonas* sp. BSi20412, which was identified only in sample number 1, was aligned with those of *Escherichia coli*, and *Bacillus subtilis* to detect species-specific sequences. The primer set for the first nested PCR reaction was designed based on unique *Marinomonas* sp. BSi20412 sequences. The second primer set was designed within the first primer set. The specificity of these primer sets was examined using the same template DNA that was applied to the microarrays. All of the samples were examined with *E. coli* genomic DNA as a positive control and distilled water as a negative control (N.C.). Pan-prokaryotic primer set was used to demonstrate the existence of prokaryotes DNA in the samples.

To identify habitat-specific patterns of microflora at the species level, hierarchical clustering analysis was performed for the top 200 sequences found in each sample as described in the Experimental Section. As a result, seven clusters were identified that were specific to each sample (Figure 3a). In cluster 2 (Tonegawa River), bacteria were detected that are not indigenous to the environment but are isolated from domestic animals or humans, such as *Mycoplasma conjunctivae* HRC/583, *Actinomyces hyovaginalis*, and *Campylobacter hominis*, which is consistent with the fact that there are many cattle farms upstream of this river (Figure 3b). In contrast, sequences in cluster 1 (Nemuro, northern part of Japan) included some identified at high latitude, such as *Pseudoalteromonas* sp. BSi20301 and BSi20493 and *Marinomonas* sp. BSi20412 detected in Arctic Sea samples [18] (Figure 3c). Nested PCR analysis confirmed that *Marinomonas* sp. BSi20412 was detected only in sample 1 (Figure 4). 

**Figure 5:**
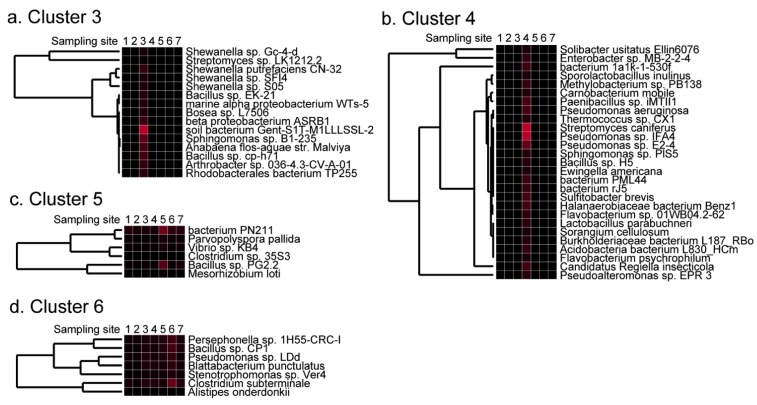
Clusters that had strong signals only in particular samples. The detail of cluster 3 (**a**), 4 (**b**), 5 (**c**), and 6 (**d**) identified in Figure 3a was shown with the name of species.

In contrast, sequences coding for thermophilic bacteria, such as Alpha proteobacterium HTA473 isolated from the Mariana Trench [19], or *Thermus* sp. HR13 isolated from a hot spring [20] were found only in cluster 7 (Yoron, the southern part of Japan) (Figure 3d). A detailed listing of species that generated strong signals in clusters 3–6 are shown in Figure 5, and the most frequently detected species in each sample are shown in Table S2. These data indicate the possibility that some of the prokaryote species can be geo-located using DNA microarrays and cluster analysis, and that the process might depend on the marine current, temperature, and human activities.

### 2.5. Discussion of Results

We designed a novel DNA microarray that can theoretically be specific to approximately 64,000 prokaryotic species in the database, and evaluated it by a comprehensive analysis of microflora in the environmental water around Japan. Previous metagenomic analyses of water in Japan only studied specific conditions such as subsurface or geothermal water in gold mines [21,22] or deep sea hydrothermal sites [23], or only searched for specific taxonomic groups [24]. We collected environmental water from north to south of Japan, and used cluster analysis to reveal several unique groups of species among the samples. A cluster specific to sample 2 contained three pathogenic bacteria. It is possible that these bacteria are derived from sewage or cattle farms, because the collection site was downstream of the Tonegawa River, which has a large amount of livestock production in its basin [25].

The microarray also detected potentially “pathogenic” bacteria; however, this method cannot immediately evaluate if they were actually pathogenic or even if they were viable. For example, *Campylobacter jejuni*, *Escherichia coli*, and *Klebsiella pneumoniae* can exist in a viable but non-culturable (VNC) state in the environment [26]. The pathogenic bacteria identified in this study (Table 2) are known indigenous bacteria that cause waterborne diseases [27,28]. Therefore, data from this DNA microarray might be able to provide information that can be related back to the number of such pathogens and risk of infection. It will also be useful for cases of *Clostridium perfringens* [29,30], in which it will be suspected that most contamination originates from human activities like sewage or livestock production facilities [31]. Any relationship between these pathogenic bacteria and diseases or food poisoning around their sampling sites would have to be independently evaluated to verify actual pathogenicity.

Among the metagenomic analyses of water microflora, previous studies have reported the correlation of its composition with temperature [26,32]. The cluster that had unique signals in sample 7 (Yoron) contained bacteria that are found in warmer environments. Together with *Vibrio cholerae*, the bacteria in that specific cluster may be useful in evaluating changes in microflora caused by the influence of warmer marine temperatures. This hypothesis needs to be confirmed by studying more materials obtained from multiple sampling points having similar environmental temperature. Specific signals were also detected in sample 1 (Nemuro), which was collected from a lower temperature environment. Three of the species in this sample (*Pseudoalteromonas* sp. BSi20301 and BSi20493, and *Marinomonas* sp. BSi20412) were detected in a study of Arctic Sea samples from Canada [18]. Therefore, the decreases of signal intensities against these probes may indicate the progress of marine warming. Moreover, nested PCR revealed the presence of *Marinomonas* sp. BSi20412, which is an easier assay than microarray analysis. Prokaryotes that belong to cluster 1 may be analyzed simultaneously for a more precise index of global warming. Thus, it is possible that this microarray can distinguish prokaryotic species that are applicable to the analysis of environmental microflora, with results comparable to those obtained by pyrosequencing [33]. Determination of the bacterial index that reflects environmental changes will provide a tool to assess global warming. In our ongoing studies we are analyzing more samples from the same and different sampling points for several years. Using these approaches it may be possible to find specific index species that represent particular environments, and/or to illustrate the characteristics of each environment in an easily recognizable ways, such as by self-organization maps.

## 3. Experimental

### 3.1. DNA Microarray Design

Microarray probes were designed from 142,860 sequences of 16S rRNA released on 24 January 2009 by the DDBJ database [34]. In order to generate the most specific probes to each sequence, 24 nucleotides (nts) of each sequence were first searched at 1-bp intervals as an initial pool of candidate probes, and then applied filters based on homology against all other sequences in the 16S rRNA database [35]. In the filtering process, scores of each 24-mer oligo were calculated for the average 15-mer frequency in the genome, the oligo frequency in the database, and uniqueness. The 15-mer frequency score was used to avoid probes in repetitive regions while the oligo frequency score was used to identify the number of exact matches within the database. The uniqueness information was reduced to a Boolean value based on the weighted mismatch score. To be considered unique, a 24-mer oligo must have a weighted mismatch score greater than 10. The *Tm*, base pair composition, and self-complemetarity were also part of the probe selection parameters. 

Finally, one to three different 29-mer probes were designed for each DNA sequence in the database. As a result, most probes were located within 5' variable region. Eventually, a total of 258,697 specific probes against 97,927 sequences were designed. It was not possible to design specific probes for the rest of 44,933 16S rRNA sequences in the database, since all their candidate probes had failed the filtering criteria. The 97,927 sequences included 72,233 sequences in which highly stringent probes were designed, and other 25,694 sequences with rather loose probes. 

The sequences to which only loose probes were made were also included in the array, since the definition of the stringency was calculated based on the sequence in the database that covers only a few percent of the bacterial species in the nature. Therefore, DNA microarray may not determine specific species because there are many unidentified species that may have homologous sequences. However, it will be useful to represent gross characteristics and changes of particular environment. Designed sequence-specific probes with NimbleGen sample tracking control probes (Roche) and random probes were mounted to the array chip in duplicate. 

Duplicate probes were distributed in a random fashion on the array chip and utilized for the evaluation for accuracy (see Section 3.5 Data Analysis of DNA Microarray). This prokaryotic microarray was theoretically expected to identify about 64,000 bacterial and archaeal species in the database. Data from probes designed in the same species were treated as independent data.

### 3.2. DNA Extraction from Environmental Water

One liter of environmental water was passed through a 0.22-μm filter and DNA was extracted from the filter using the Ultraclean Water DNA Kit (Mo Bio Laboratories Inc., Carlsbad, CA, USA) according to the manufacturer’s guidelines. Only clear water with no visible turbidity was sampled. Briefly, the membrane was cut into small pieces and vortexed with 5 g of microbeads and 4 mL of bead solution for 30 s in a 15-mL sterile test tube. The addition of 0.5 mL of solution WD1 was followed by another 30-s vortex. The tube was then set horizontally and further vortexed for 10 min. After centrifugation at 2,500 ×*g* for 1 min, the supernatant was transferred to a new tube, mixed with 0.6 mL of solution WD2, and incubated at 4 °C for 5 min. After centrifugation at 2,500 ×*g* for 4 min, the supernatant was transferred to a new tube and mixed with 8 mL of solution WD3. This mixture was loaded into a spin filter tube and centrifuged at 2,500 ×*g* for 2 min. After discarding the flow through, 3 mL of solution WD4 was added, and the spin filter was centrifuged at 2,500 ×*g* for 3 min. The flow through was discarded before the filter was centrifuged at 2,500 ×*g* for 5 min. After transferring the spin filter to a new tube, DNA was recovered by adding 3 mL of solution WD5 and centrifuging at 2,500 ×*g* for 2 min, followed by ethanol precipitation.

### 3.3. Whole Genome Amplification (WGA)

Recovered metagenomic DNA was uniformly amplified using WGA in order to perform fluorescent labeling and for further PCR analysis. Although non-uniform amplification is noted in some WGA applications, we used Genomeplex Whole Genome Amplification Kit (Sigma, St. Lois, MO, USA) based on the reported comparative study [36]. We confirmed in our previous study that the results of this WGA gave superior detection of trace amounts of DNA in subsequent PCR detection [37]. Briefly, 10-μL of DNA was treated with 1 μL fragmentation buffer at 95 °C for 4 min and chilled on ice. Library preparation buffer and library stabilization buffers (1 μL of each) were added to the samples. The solution was incubated at 95 °C for 2 min and chilled on ice. After the addition of 1 μL of Library Preparation Enzyme, the DNA was sequentially incubated at 16 °C for 20 min, 24 °C for 20 min, 37 °C for 20 min, and 75 °C for 5 min. The solution was amplified with 7.5 μL of Amplification Master Mix, 47.5 μL of distilled water, and 5 μL (12.5 units) of Jumpstart Taq DNA polymerase under the following conditions: initial denaturation at 95 °C for 3 min, 14 cycles of 94 °C for 15 sec and 65 °C for 5 min. 

### 3.4. Fluorescent Labeling, Hybridization and Analysis of DNA Microarray

The 16S rRNA sequences in the sample were fluorescently labeled using 10-mer primers designed against conserved prokaryotic sequences [38,39,40]. Four Cy3-labeled reverse primers and three unlabeled forward primers were used simultaneously to label sample DNA for hybridization (Table S3). The primers were located in the 5' variable region of the 16S rRNA gene, where most of the array probe sequences were located (Figure S2). The labeling and hybridization protocols were modifications of the method used by Akama *et al*. [41]. Cy3 labeling reactions were performed with the NimbleGen One-color DNA Labeling Kit (Roche NimbleGen, Inc. Madison, WI, USA): 1 µg DNA was incubated for 10 min at 98 °C with 1 OD unit of 16S rRNA-specific primers. A random nonamer was used as the control primer. The addition of 8 mmol of dNTPs and 100 U of Klenow fragment (New England Biolabs, Ipswich, MA, USA) was followed by incubation at 37 °C for 2 h. The reaction was stopped by adding 0.1 volume of 0.5 M EDTA, and the labeled DNA was precipitated with isopropanol and resuspended in NimbleGen Hybridization Buffer (Roche NimbleGen). The Cy3-labeled samples were denatured at 95 °C for 5 min and hybridized to the arrays in a NimbleGen Hybridization System (Roche NimbleGen) for 18 h at 42 °C. The arrays were washed using the NimbleGen Wash Buffer Kit (Roche NimbleGen), dried by centrifugation, and scanned at a 5-µm resolution using a GenePix 4000B scanner (Molecular Devices, Sunnyvale, CA, USA) and NIMBLESCAN 2.5 software (Roche NimbleGen) to obtain fluorescence intensities. Accuracy of hybridization was assured by internal controls spotted in duplicates, and reproducibility of array results was confirmed in preliminary studies by analyzing the same sample twice.

### 3.5. Data Analysis of DNA Microarray

Raw signal intensities were first corrected based on background subtraction methods proposed by Xie *et al*. [42], by which most signals were shifted close to zero (Figure S3). Remaining noise was further evaluated by setting several threshold levels based on *p*-values. MAS5.0 background method from Affymetrix [43] was also applied. Data from duplicate probes were treated as a pair of independent data sets and such data sets were compared to verify the reproducibility of the array. The original and corrected data were assessed by histogram, box plot and MA plot (Figure S4). 

Then, mean corrected signal intensities were calculated from the duplicate data. Because the signal intensities from each sample did not fall into a Gaussian distribution, and the number of probes that showed strong signals in each samples were about 1,000 (Figure S5), we decided to analyze these top 1,000 sequences in each sample for taxonomic classification and detection of pathogenic bacteria. Reliability of the data was evaluated by comparing the data from duplicate probes in each sample to create heat maps and perform clustering for phylum, class, order, family, genus and species, and to calculate *p*-values based on probability distribution (data not shown). 

A total of 743 non-redundant sequences that were included in the top 200 sequences in each of the seven samples were subjected to further hierarchical cluster analysis. The rank of the signal intensity in each microarray was changed to the reciprocal number and analyzed with Cluster 3.0 [44]. Briefly, in the beginning of the hierarchical cluster analysis each element (data) was in a cluster of its own. The clusters were then sequentially combined into larger clusters, until all elements end up being in the same cluster. At each step, the two clusters separated by the shortest distance were combined. The definition of “shortest distance” was calculated using Lance-Williams algorithms (Ward’s method). Java Treeview 1.0 [45] was then used for tree visualization of the microarray data.

### 3.6. Nested PCR

The 16S rRNA sequence of *Marinomonas* sp. BSi20412 (GenBank Accession No. DQ537503) was aligned with those of *Escherichia coli* (GenBank Accession No. J01859) and *Bacillus subtilis* (GenBank Accession No. AY219900) to identify a *Marinomonas-*specific sequence. In this region, the following PCR primer sets were designed for nested PCR: 5'-TTCAGGGGTGAGGAAGGGCGTTTG-3' (initial forward); 5'-CTCACAGTTCCCGAAGGCACTCCA-3' (initial reverse); 5'-ACCCAGATGTCTTGACGTTAGCCC-3' (second forward); 5'-GCATCTCTACCGGATTCTGTGGA-3' (second reverse) to amplify a 242-bp fragment. Touchdown PCR was performed with the external primers and the same template DNA that was used for microarray analysis. The PCR product was diluted and used as the template for the second round of nested PCR as described previously [37]. The primer set that could amplify all of the prokaryotic 16S rRNA sequences was used as a control: 5'-CCTACGGGAGGCAGCAG-3' (forward); 5'-ATTACCGCGGCTGCTGG-3' (reverse) to amplify a 161-bp fragment [46]. 

## 4. Conclusions

In conclusion, we have developed a 16S rRNA microarray that allows comprehensive detection of prokaryotes in a simple and cost-effective manner, making it possible to analyze clusters that include both known and novel index species. This DNA microarray will be a useful tool in the detection of pathogenic and temperature-sensitive bacteria in environmental samples. 

## Supplementary Files

Supplementary File 1 Supplementary Materials (PDF, 3624 KB)
