# Supplementary material for: Comprehensive Analysis of Prokaryotes in Environmental Water Using DNA Microarray Analysis and Whole Genome Amplification
Source: Pathogens. 2013 Oct 30;2(4):591–605. doi: 10.3390/pathogens2040591 (PMC4235703; doi:10.3390/pathogens2040591)

Supplementary Materials

Comprehensive Analysis of Prokaryotes in Environmental Water Using DNA Microarray Analysis and Whole Genome Amplification

Table S1. Original probe composition and array data in phylum level.

Phylum	Probe composition (No.)	Probe composition (%)	Nemuro (%)	Tonegawa river (%)	Shimane-kaka (%)	Hachijo (%)	Kagoshima (%)	Ikeda lake (%)	Yoron (%)
Acidobacteria	183	0.19	0.4	0.1	0.2	0.5	0.2	0.2	0.1
Actinobacteria	17,415	17.83	13.2	15.2	8.7	7.5	8.3	7.4	12.8
Aquificae	150	0.15	0.5	0.5	0.7	0.7	0.7	0.7	0.5
Bacteroidetes	5,223	5.35	6.8	6.4	6.1	7.4	8.3	7.8	6.6
Chlamydiae	147	0.15	0.2	0.3	0	0	0	0	0
Chlorobi	175	0.18	0.2	0.3	0.2	0.3	0.4	0.2	0.3
Chloroflexi	95	0.10	0.2	0.2	0.2	0.2	0.2	0.1	0.1
Cyanobacteria	2,957	3.03	3.4	2.5	3.2	3.1	4.0	2.9	2.6
Deinococcus-Thermus	433	0.44	0.3	0.3	0	0	0.1	0	0.1
Fibrobacteres	58	0.06	0.1	0	0	0.1	0	0	0
Firmicutes	19,008	19.46	17.4	17.3	15.3	17.1	16.4	11.9	18.6
Nitrospirae	101	0.10	0.1	0	0	0	0.1	0.1	0.1
Planctomycetes	163	0.17	0.2	0.1	0	0	0	0	0
Proteobacteria	40,137	41.09	47.5	47.6	56.1	53	51.4	59.7	48.8
Spirochaetes	1,008	1.03	1.0	0.9	1.7	0.9	1.1	0.8	1.1
Synergistetes	48	0.05	0.2	0	0	0	0	0.1	0
Tenericutes	1,884	1.93	1.2	1.0	1.2	1.2	1.1	0.8	0.9
Thermotogae	102	0.10	0.3	0.4	0.3	0.3	0.4	0.4	0.4
Verrucomicrobia	100	0.10	0	0	0.1	0	0	0	0
unidentified/unclassified	6,564	6.72	6.4	6.3	6.0	7.4	7.0	6.8	6.7
Crenarchaeota	219	0.22	0.1	0.1	0	0.1	0.1	0	0.1
Euryarchaeota	1,511	1.55	0.3	0.5	0	0.2	0.2	0.1	0.2
Total	97,681	100	100	100	100	100	100	100	100

Table S2. Most frequently detected species in each sample.

Nemuro	Tonegawa river	Shimanekaka	Hachijo	Kagoshima	Ikeda lake	Yoron
Enterococcus faecium	*Enterococcus faecium*	*Lactobacillus fermentum*	*Bacillus* sp. *TB1*	*Streptomyces* sp. *334F01*	*Rhizobium tropici*	*Staphylococcus* sp. *RCTIC-P62*
Streptomyces sp. *334F01*	*Rhizobium* sp. *STM 4O41*	*Bacillus* sp. *th2*	*Bacillus firmus*	*Bacillus thuringiensis*	*Firmicutes bacterium MMD12*	*Bacillus* sp. *J357*
Bradyrhizobium lupini	*soil bacterium T8*	*Rhizobium* sp. *STM 4O41*	*Lactobacillus sanfranciscensis*	*Lactobacillus sanfranciscensis*	*Helicobacter* sp. *WB1F*	*Lactobacillus fermentum*
Bacillus firmus	*Streptomyces* sp. *334F01*	*freshwater bacterium FO-121*	*Lactobacillus plantarum*	*Rhizobium* sp. *STM 4O41*	*Gram-negative bacterium cL-10-26*	*Sphingobacterium multivorum*
Bacillus thuringiensis	*Sphingomonas jaspsi*	*Pantoea* sp. *BD 309*	*Rubrobacter xylanophilus*	*Erythrobacter* sp. *JL990*	*Bacillus* sp. *th2*	*Elbe River snow isolate IsoC2*
Rhizobium sp. *STM 4O41*	*Curtobacterium albidum*	*Bacillus* sp. *J357*	*Bradyrhizobium lupini*	*Sphingomonas paucimobilis*	*Lactobacillus fermentum*	*bacterium UASWS0091*
Sandal spike phytoplasma	*Curtobacterium albidum*	*Sphingobacterium multivorum*	*Staphylococcus* sp. *ARCTIC-P62*	*Enterococcus faecium*	*Treponema* sp. *1:4:292*	*Helicobacter pylori*
soil bacterium T8	*Lactobacillus sanfranciscensis*	*Lactobacillus sanfranciscensis*	*Curtobacterium albidum*	*soil bacterium T8*	*soil bacterium T8*	*Rhizobium tropici*
Sphingomonas jaspsi	*Bacillus* sp. *TB1*	*Enterococcus faecium*	*Rhizobium* sp. *STM 4O41*	*Rubrobacter xylanophilus*	*Pantoea* sp. *BD 309*	*freshwater bacterium FO-121*
Curtobacterium albidum	*Erythrobacter* sp. *AS-25*	*Rhizobium* sp. *STM 4O44*	*Acinetobacter* sp. *BR-12*	*Brachybacterium paraconglomeratum*	*Pseudomonas* sp. *ps11-30*	*Streptomyces* sp. *334F01*
Staphylococcus sp. *ARCTIC-P62*	*Natranaerobius thermophilus*	*Rhizobium tropici*	*Rhizobium tropici*	*Streptomyces* sp. *JXNT-A-22*	*Brucella* sp. *CGL-1*	*Enterococcus faecium*
Bacillus sp. *TB1*	*Rhizobium tropici*	*Helicobacter* sp. *WB1F*	*Streptomyces* sp. *334F01*	*Bacillus pumilus*	*Candidatus Midichloria mitochondrii*	*Brachybacterium paraconglomeratum*
freshwater bacterium FO-121	*Sandal spike phytoplasma*	*Acinetobacter* sp. *BR-12*	*Sphingomonas jaspsi*	*Candidatus Phytoplasma AZ04-1-5*	*freshwater bacterium FO-121*	*Klebsiella* sp. *MSI78*
Bacillus sp. *th2*	*Rhodococcus* sp.	*Natranaerobius thermophilus*	*freshwater bacterium FO-121*	*Holospora obtusa*	*bacterium UASWS0091*	*Rhizobium* sp. *M061122-8*
Paenibacillus sp. *SKU 11*	*Bacillus* sp. *th2*	*Streptomyces* sp. *334F01*	*gamma proteobacterium RBE2CD-47*	*Pseudomonas* sp. *R-35697*	*Psychrobacter pulmonis*	*soil bacterium T8*
Bacillus sp. *J357*	*Lactobacillus plantarum*	*Staphylococcus* sp. *ARCTIC-P62*	*Erythrobacter* sp. *JL990*	*Bacillus* sp. *TB1*	*Acinetobacter* sp. *BR-12*	*Pseudomonas* sp. *R-35697*
Pantoea sp. *BD 309*	*Lactobacillus fermentum*	*gamma proteobacterium AE060401_3*	*Bacillus* sp. *J357*	*Pseudoalteromonas* sp. *ST061013-057*	*Rhizobium* sp. *STM 4O44*	*Sandal spike phytoplasma*
Pseudomonas sp. *MT27*	*Psychrobacter pulmonis*	*Lactobacillus plantarum*	*Pseudomonas* sp. *R-35697*	*Rhodocista xerospirillum*	*Lactobacillus sanfranciscensis*	*Sphingomonas paucimobilis*
Rhizobium tropici	*Brachybacterium paraconglomeratum*	*Clostridium perfringens*	*Bacillus* sp. *th2*	*Rhodococcus* sp.	*Streptomyces* sp. *334F01*	*Mycobacterium* sp. *SSRW25-5*

Table S3. Labeling primers for 16S rRNA microarray.

	16S rRNA position [a]	Strand	Sequence
Cy3-labeling	355	Reverse	GCTGCCTCCC
	519		ATTACCGCGG
	1393		TACAAGACCC
	1458		TTGTTACGAC
Non-labeling	35	Forward	ATCCTGGCTC
	778		TCCACGCTGT
	972		CGAAGAACCT

[a] Positions are referenced against the *E. coli* 16S rRNA sequence.

Figure S1. Microarray data classified at the class, order, family, genus and species levels. Distribution was illustrated by different colors, but not names.

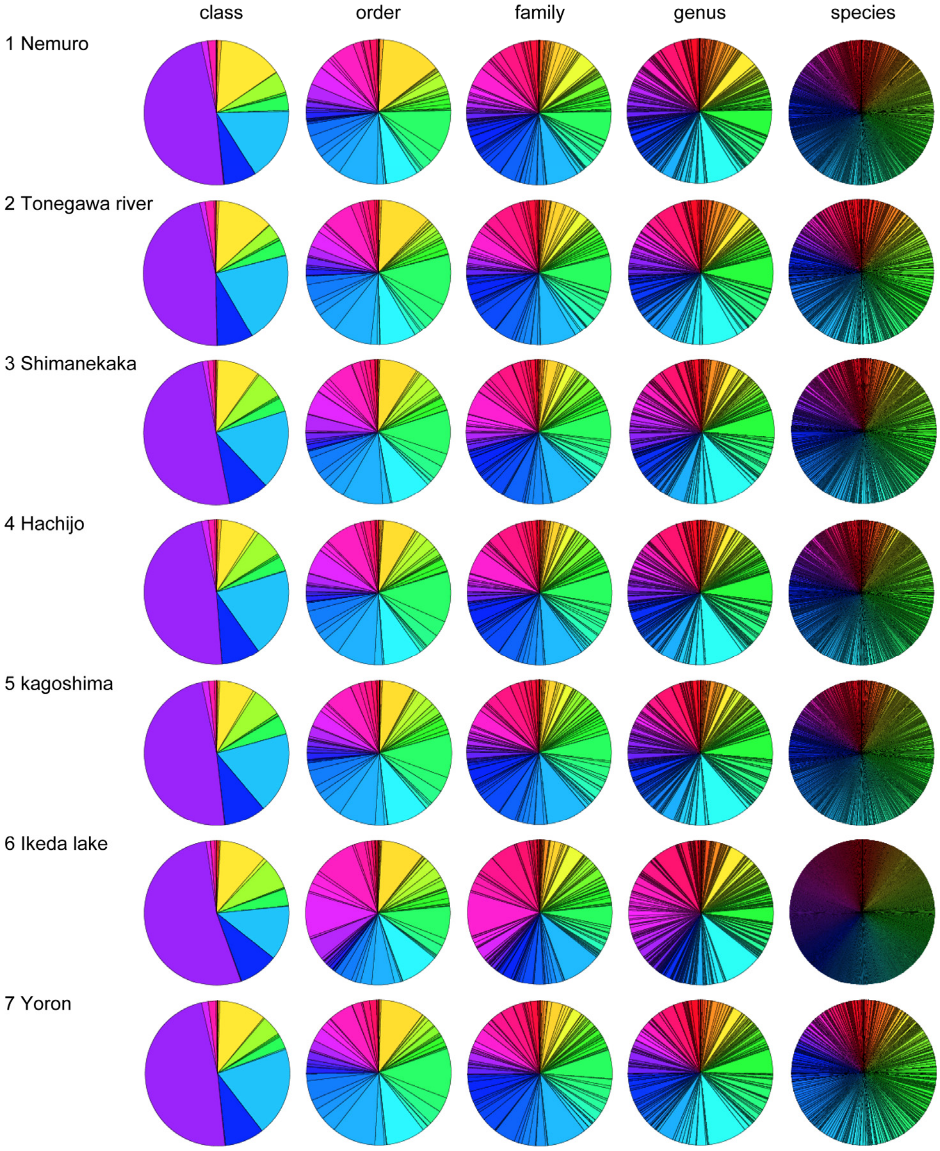

Figure S2. Variable regions and labeling primer sites in 16S rRNA. Three unlabeled forward primers and four Cy3-labeled reverse primers covering 16S rRNA variable region one to nine were used to label the sample sequences. Cy3 was labeled at the 5' end of the reverse primers, which are the reverse strand against the array probes.

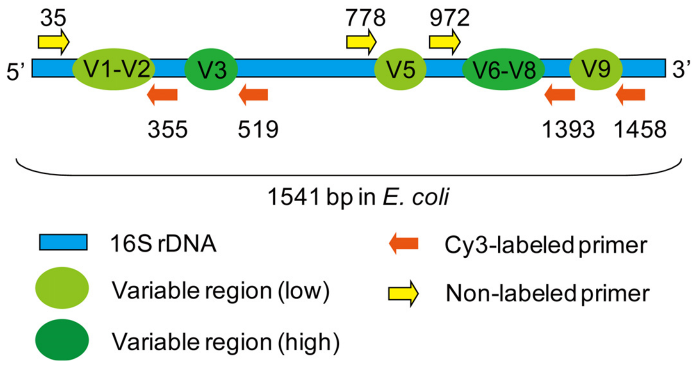

Figure S3. (**a**) Histogram of original signal intensities; (**b**) corrected intensities after background subtraction [39]. Two independent data sets from duplicate probes completely overlapped in the graph, verifying the high reproducibility of this array. Although original signal intensities were variable in low intensities, most weak signals came very close to zero after background subtraction.

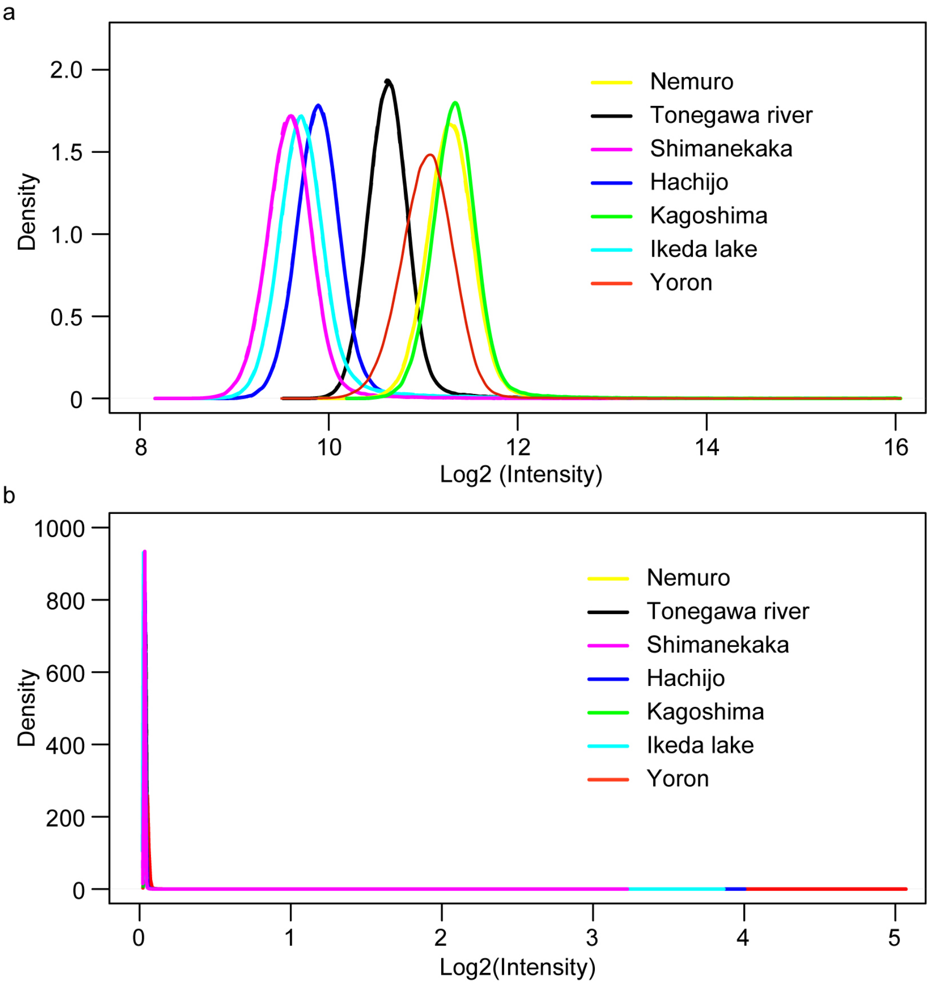

Figure S4. MA plot of each sample before and after background correction. Two sets of data from duplicate probes were treated as independent data sets. After correction, intensities of most probes came close to zero. Loess curve became almost a straight line (M = 0). All the interquartile range (IQR) became small.

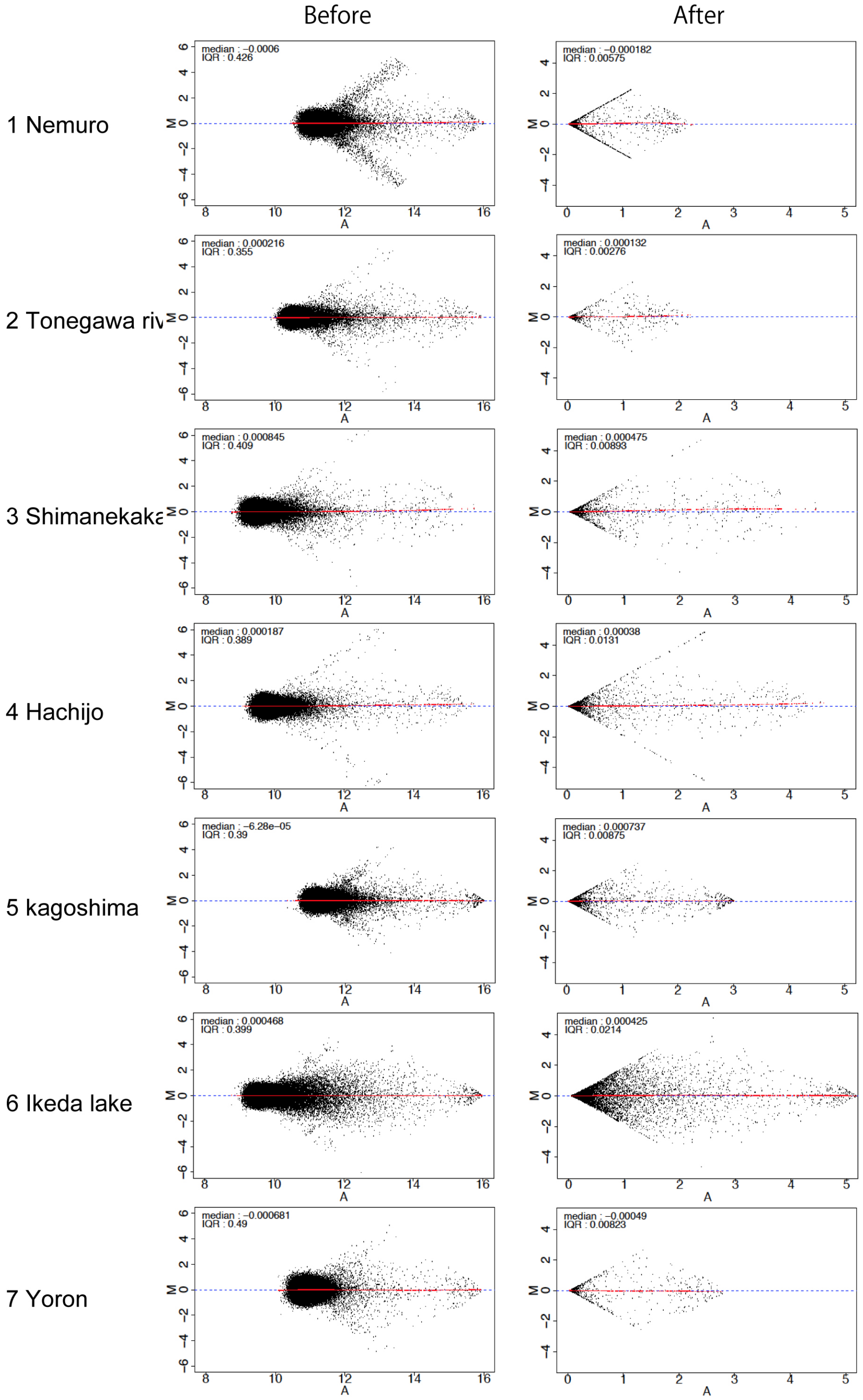

Figure S5. Histogram of positive signal intensities obtained with the microarray after normalization. Signal intensities shown as 2^n on x-axis and probe number shown as 10^n on y-axis were plotted. Dashed line shows position of the sequence with the 1,000th strongest signals in each sample.

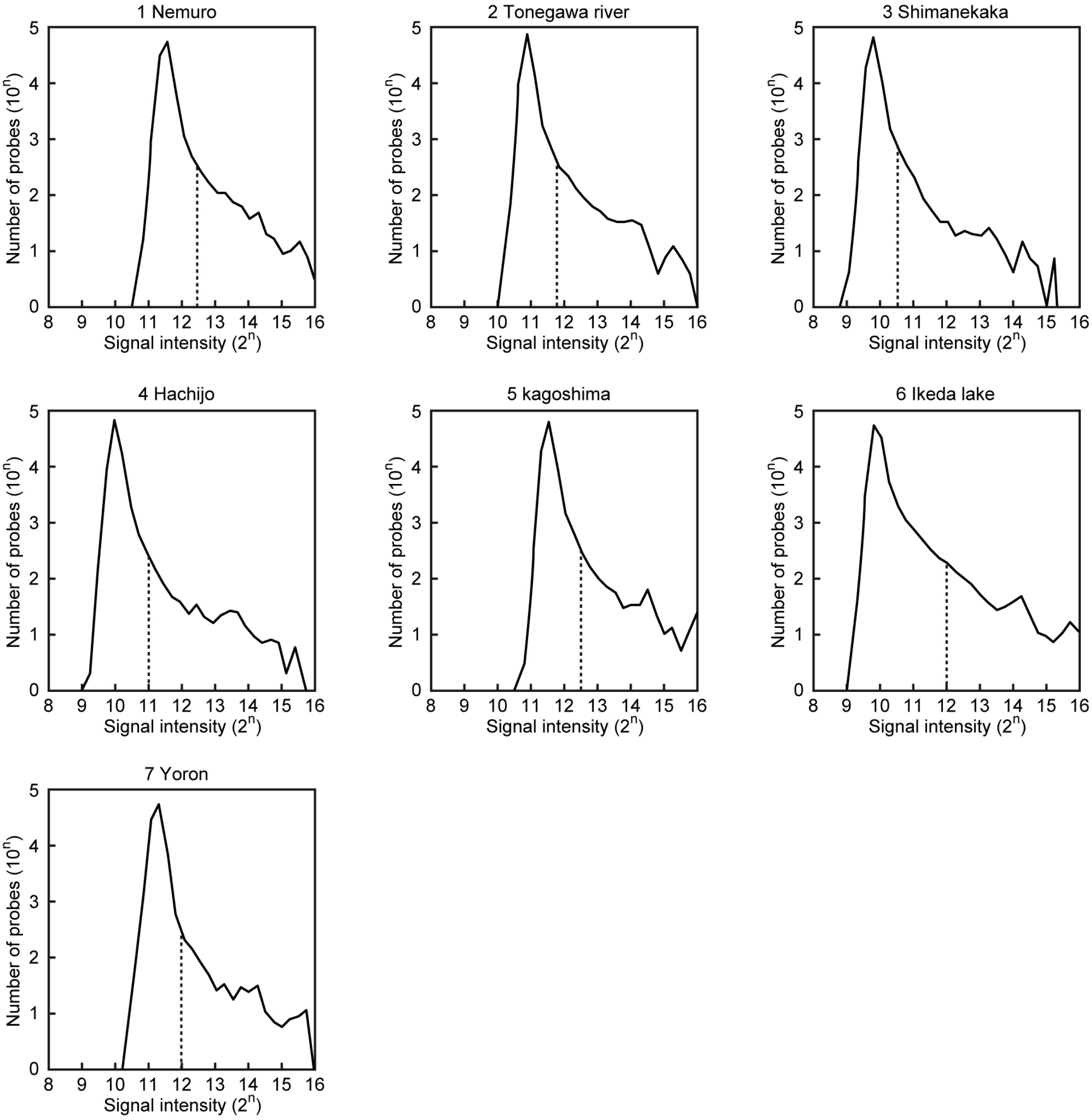

Supplement: Supplementary File 1 — Supplementary Materials (PDF, 3624 KB) [file pathogens-02-00591-s001.pdf]
